# Impact of Atmospheric Microparticles on the Development of Oxidative Stress in Healthy City/Industrial Seaport Residents

**DOI:** 10.1155/2015/412173

**Published:** 2015-04-30

**Authors:** Kirill Golokhvast, Tatyana Vitkina, Tatyana Gvozdenko, Victor Kolosov, Vera Yankova, Elena Kondratieva, Anna Gorkavaya, Anna Nazarenko, Vladimir Chaika, Tatyana Romanova, Alexander Karabtsov, Juliy Perelman, Pavel Kiku, Aristidis Tsatsakis

**Affiliations:** ^1^Vladivostok Branch of the Far Eastern Center of Physiology and Pathology of Respiration, Institute of Medical Climatology and Rehabilitative Treatment, 73g Russkaya Street, Vladivostok 690105, Russia; ^2^Far Eastern Federal University, 8 Sukhanova Street, Vladivostok 690950, Russia; ^3^Far Eastern Center of Physiology and Pathology of Respiration, 22 Kalinina Street, Blagoveshchensk 675000, Russia; ^4^Far Eastern Geological Institute FEB RAS, 159 Prospekt 100-letiya, Vladivostok 690022, Russia; ^5^Department of Toxicology and Forensics, Medical School, University of Crete, Heraklion, 71300 Crete, Greece

## Abstract

Atmospheric microsized particles producing reactive oxygen species can pose a serious health risk for city residents. We studied the responses of organisms to microparticles in 255 healthy volunteers living in areas with different levels of microparticle air pollution. We analyzed the distribution of microparticles in snow samples by size and content. ELISA and flow cytometry methods were employed to determine the parameters of the thiol-disulfide metabolism, peroxidation and antioxidant, genotoxicity, and energy state of the leukocytes. We found that, in the park areas, microparticles with a size of 800 *μ*m or more were predominant (96%), while in the industrial areas, they tended to be less than 50 *μ*m (93%), including size 200–300 nm (7%). In the industrial areas, we determined the oxidative modification of proteins (21% compared to the park areas, *p* ≤ 0.05) and DNA (12%, *p* ≤ 0.05), as well as changes in leukocytes' energy potential (53%, *p* ≤ 0.05). An increase in total antioxidant activity (82%, *p* ≤ 0.01) and thiol-disulfide system response (thioredoxin increasing by 33%, *p* ≤ 0.01; glutathione, 30%, *p* ≤ 0.01 with stable reductases levels) maintains a balance of peroxidation-antioxidant processes, protecting cellular and subcellular structures from significant oxidative damage.

## 1. Introduction

The development of the research direction of city ecology was caused by the global challenges which humanity is facing due to the increasing industrial activity. Furthermore, the world urban population continues to grow and so does the prevalence of environmental diseases. City air is the first marker of total environmental pollution. Atmospheric pollution in general is one of the leading factors related to health risks in cities [1–3]. Of all atmospheric particles, microsized particles are the most dangerous [4], especially those smaller than 10 *μ*m. Such particles slowly disappear from the atmosphere and have a relatively long life in a suspended state. They significantly accumulate in the environment even at a considerable distance from the sources of pollution. Unlike gases, microparticles are heterogeneous complex mixtures consisting of numerous components and their properties vary widely in terms of time and space [5].

It has been shown that long-term exposure to high levels of particulate matter <2.5 *μ*m in aerodynamic diameter (PM_2.5_) in the atmosphere reduces life expectancy among the population from several months to a few years [6]. In addition, suspended particles absorb a large amount of toxic substances that can enter an organism [7].

The impact of microsized particles on a living organism is fundamentally different from that of large-scale pollutants [4]. One of the main mechanisms of toxic action by microparticles is linked with their ability to induce the production of reactive oxygen species (ROS). Moderate levels of ROS modulate cell signaling, proliferation, and gene expression. The increase in ROS levels reduce the cellular antioxidant capacity, destroy the cell antioxidant defense system, and lead to oxidative stress development [8]. Oxidative stress causes direct or indirect damage to key cell components, such as lipids, proteins, and nucleic acids, and also inhibits DNA repair [9]. Cell competency in resisting pathogenic microsized xenobiotics mainly depends on the efficiency of the individual repair mechanisms.

Redox systems are one of universal intracellular units mediating ROS regulatory functions. They are presented mainly by glutathione (GSH) and thioredoxin (TRX) [10], the most sensitive to oxidative stress systems. They effectively restore disulfide bonds in proteins through the activation of specific enzymatic systems. For the glutathione system, this is NADPH-dependent glutathione reductase and, for the TRX system, NADPH-dependent TRX-reductase (TrxR). By modulating the transcription factors' activity, particularly in the case of NF-*κ*B, the TRX system plays a key role in the protection of the cell components from the destructive effect of various manifestations of oxidative stress [11]. Different TRX system agents affect the activity of DNA repair endonuclease according to shifts in the cell redox balance [12].

TRX and GSH systems participate in the regulation of proinflammatory cytokines expression and apoptosis [13, 14]. In addition to its function as a key regulator of the oxidative stress-associated redox processes, thioredoxin plays an important role in protein folding, the regulation of nitrosative stress [15], cell growth, and differentiation [13, 15, 16]. Interaction between glutathione and thioredoxin redox cell systems proceeds in particular through thioredoxin glutathionylation [17, 18].

At the end of the last century (1979), suspended particles were included within a number of polluting substances considered within the “Convention on long-range transboundary air pollution” by the Economic Commission for Europe of the United Nations (UNECE), whose tasks include the quality management of atmospheric air, as well as the regulation and control of emissions of polluting substances in the atmosphere in Europe.

The impact of atmospheric microsized particles on the urban population under different environmental loads and the assessment of the organisms' response are important research problems in this new century.

Based on all the mentioned above the aim of the present research was to assess the contents and qualitative structure of atmospheric microparticles and their role in the development of oxidative stress in healthy residents of the industrial city.

## 2. Methods and Materials

### 2.1. Area and Particles Sample Collection

Vladivostok, the largest city in the Far Eastern Federal District of Russia and the largest Russian seaport on the Pacific Ocean, is located in the southern extremity of a peninsula that juts far out into the sea. In Vladivostok, there are relatively few industrial enterprises, but the road traffic is quite intensive. This determined our choice of it as an object for studying a background of natural and anthropogenic atmospheric suspensions in the city and its suburban area.

Snow samples were collected during snowfall periods from 2010 to 2014 in 13 districts of Vladivostok [19]. The upper layer (5–10 cm) of fresh snow from a 1 m^2^ area was collected into 3 L sterile containers that had been prewashed with double-distilled water. This collection method was chosen to avoid secondary contamination by anthropogenic aerosols.

### 2.2. Particles Sample Analysis

From each sample, we took 60 mL of melted snow and analyzed it using a laser particle sizer analyzer Analysette 22 NanoTec (Fritsch, Germany). During a single measurement set, we determined the size distribution and shape of the particles.

To obtain dry samples directly from the air, we used an LSV 3.1 sampler (Derenda, Germany) with MGG filter (microglass fibre paper) with a diameter of 47 mm (Munktell, Germany). In work, we used a reference device to collect particulates from outdoor air, in compliance with the CEN 12341 (PM_10_) standard. We dried the filters prior to the measurement in the thermostat at 40°C and then weighed the filters on scales (Shimadzu AW-220, Japan) 10 times before and after the measurements.

Substantial analysis was performed using a Nikon SMZ1000 light microscope and a Hitachi S-3400N scanning electron microscope with an Ultra Dry (Thermo Scientific, USA) energy-dispersive spectrometer. The sample deposition for the electron microscopy was made with platinum.

A high resolution inductively coupled plasma mass spectroscopy (HR-ICP-MS) employing Element XR (Thermo Fisher Scientific, USA) was used to assess the Me in the collected snow PM samples. The liquid part of the samples was stored at 40°C before evaporation and analyzed according to technique CV 3.18.05-2005 [20].

Our long-term observations (2010–2014) and laser particle size, electron microscopy, and mass spectrometry analyses [19] allowed us to select 2 model points for this work from 13 studied points: the park area (the coast of Russky Island, Vladivostok suburb) and the industrial area (the largest transport hub in the city, the continental part of Vladivostok).

Studies were conducted with use of the equipment of the Interdepartmental Center of Analytical Control of a State of Environment of Far Eastern Federal University.

### 2.3. Biomedical Study Subjects and Sample Collection

The study was approved by the Committee on Biomedical Ethics and performed in 2012-2013 in Primorsky Krai (Russian Federation).

The study involved healthy residents of Vladivostok and Russky Island who had lived and worked for at least 5 years within 1 km from model areas. We surveyed 121 men and 134 women, with an age range from 28 to 54 years (mean age 37 ± 6). All volunteers were fully informed about the goals, objectives, and procedures of the study; all of them signed an informed consent form and passed the clinical and laboratory examinations. The criteria for exclusion from the study were occupational hazards, smoking, alcohol dependence, pregnancy, chronic disease (as stated in medical records or reported personally), acute illness, and/or current drug treatment (Table 1).

The participants were divided into two groups comparable by age and sex and selected according to the place of residence. Group 1 includes the inhabitants of the park area, Russky Island (one of the administrative districts of Vladivostok) and Group 2, the industrial area, the inhabitants of the continental area of the city/industrial seaport (Vladivostok) [19].

Venous blood samples (9 mL) were collected in the early morning following overnight fasting in vacuum EDTA tubes (Vacutainer). For enzyme-linked immunosorbent assay (ELISA) analyses, we centrifuged the tubes for 10 minutes on 1,200 g and collected plasma. Plasma samples were stored at −80°C until analysis. Flow cytometry analysis was performed immediately.

### 2.4. Biomedical Sample Analysis

Plasma samples were analyzed using ELISA and the colorimetric method. For the measurement, we used the reagents in Table 2.

Measurement of MMPL was done via the BD FACSCANTO II (BD Biosciences, USA) flow cytometer.

### 2.5. Statistical Analysis

Statistica 10.0 (Statsoft) software was used for the statistical analysis. All data were presented as the mean ± standard error of the mean. The differences between the groups were analyzed using the Mann-Whitney test. The correlation was analyzed using the Pearson correlation method.

## 3. Results

### 3.1. Atmospheric Suspensions in Different Areas of Vladivostok

Our long-term observations [19] made it possible to select a background for the conversion of certain city areas to the park or industrial area in relation to microparticles air pollution (Table 3).

We measured a mass fraction of suspensions (PM_10_) using sampler (Comde-Derenda, Germany), which equaled on average 0.06–0.09 mg/m^3^ for park area and 0.12–0.23 mg/m^3^ for industrial area.

#### 3.1.1. Atmospheric Suspensions in the Park Area

We considered Russky Island as a model park point. Russky Island, an administrative district of Vladivostok, is environmentally a park area; there are no large enterprises or heavy road traffic. The main influence on the composition of atmospheric suspension is caused by the sea coast (halite, silicates) and forest (organic detritus, pollen).

Natural coarsely dispersed dust prevails in samples from Russky Island, with a particle size of hundreds *μ*m, which cannot be inhalated. Such large natural particles do not penetrate into the lungs but are deposited in the upper respiratory tract and nasal cavity due to natural protective processes and thereby do not have significant negative effects on the functional state of organisms [4].

Scanning electron microscopy detected that in the samples from Russky Island the atmospheric particles contained mainly halite and aluminosilicates. Mass spectrometry of snow samples showed normal heavy metal levels.

#### 3.1.2. Atmospheric Suspensions in the Industrial Area

We considered the area of the largest transport hub as the model industrial area in the continental part of Vladivostok. Emissions of most of the industrial enterprises are produced at a height of up to 50 m. Besides industrial emissions, Vladivostok's air is polluted by smoke, ash from medium and large boilers, and combustion plants. Dust pollution exceeds the maximum permissible concentration on average in a few cases; for example, the maximum values of the air pollution vary from 1.5 mg/m^3^ in summer up to 3.4 mg/m^3^ in spring [21]. Dust prevailing in samples from industrial area has a particle size from 0.1 to tens *μ*m. Motor vehicle exhaust fumes contribute up to 51% of the air pollution in Vladivostok [21].

**Figure 1:**
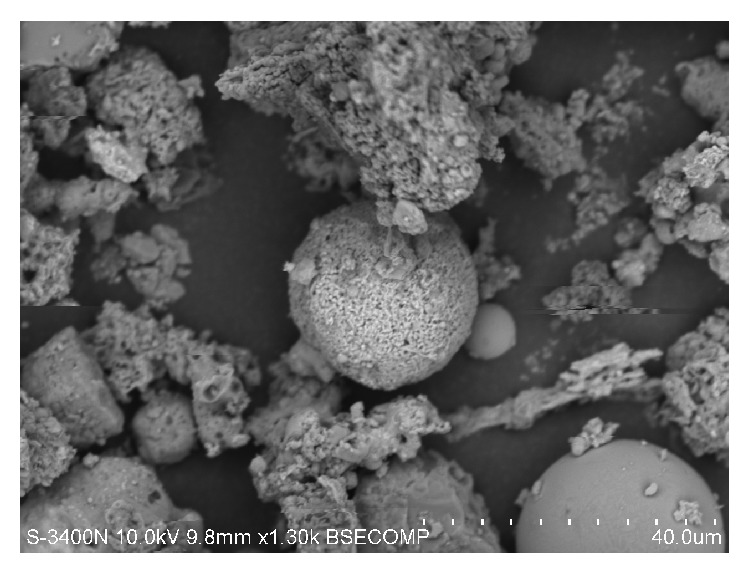
Soot and ashes particles from the industrial area of Vladivostok. Measure line: 40 *μ*m.

Data from the electron microscopic analysis show that, in the samples from the industrial area, technogenic particles originating from vehicles prevail, soot, ashes (Figure 1), metal-containing particles (Fe, Cr, Ni, Pb, and Zn), rubber, and asphalt.

In addition to large particles (Figure 1), we observed a large amount of microparticles with potentially hazardous size, 200–300 nm (7%), that were not identified in samples from the park area. These particles are mainly composed of metal and soot.

**Figure 2:**
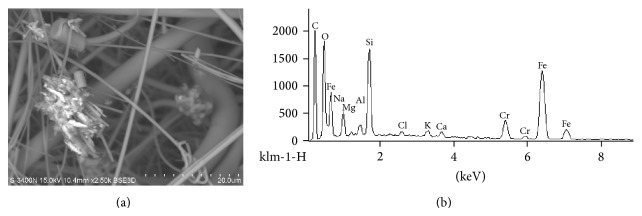
(a) Microphotograph of particles from the industrial area on sampler filter in reflected electrons. The light-colored threads are filter fibers. Measure line: 20 *μ*m. (b) Spectre of metal-containing particle (a, large light particle on the left).

As can be seen in Figure 2(b), a typical polymetallic particle is composed of Fe and Cr. The particle is aggregated and contains smaller, needle-like components.

**Figure 3:**
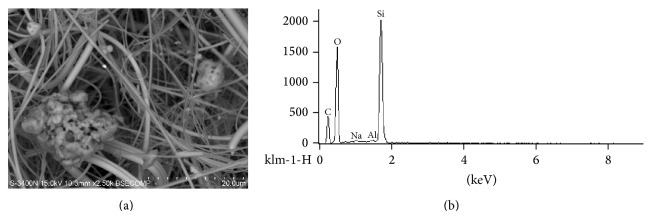
(a) Microphotograph of particles from the park area on the sampler filter in the reflected electrons. The light-colored threads are filter fibers. Measure line: 20 *μ*m. (b) Spectre of a metal-containing particle (a, the large grey particle in the left).

As can be seen in Figure 3(b), a typical mineral particle is composed of Si and Al. The particle is aggregated and contains smaller, ball-like components.

The chemical composition of snow in Vladivostok, analyzed by mass spectrometry, shows that the Cr content increased in the industrial area (5.826 ± 1.7 versus 0.35 ± 0.2 in the park area, *p* ≤ 0.001).

### 3.2. Blood Parameters in Healthy Residents in Different Areas of Vladivostok

The measured blood parameters are presented in Tables 4 and 5.

We found increased protein (21% compared to the park area) and DNA (12%) oxidative damage level and significantly increased (53%) levels of leukocytes with reduced mitochondrial membrane potential in healthy residents of the industrial area of the city/industrial seaport. The increasing MDA level (41%) and MDA/AOA ratio (29%) indicate the activation of free radical peroxidation and shifting balance in the lipid peroxidation-antioxidant defense (LPO-AOD) system towards peroxidation.

The one of the main mechanisms of negative health effects caused by atmospheric microparticles is linked with their ability to induce the production of ROS and the oxidative stress [22, 23]. Microparticles can penetrate more deeply into the respiratory tract than larger particles [4, 24]. In addition, microparticles can adsorb toxic substances, including metals [7]. The increase in the levels of prooxidant markers (MDA, 8-OHdG, PC) in residents of an industrial area comparing to park areas (Table 4) is possibly linked to differences in the content of transition metals in particles (Figures 2 and 3). These metals enhance the generation of ROS, promoting via Fenton or Haber-Weiss reaction [25] the formation of highly reactive hydroxyl radicals that can react with almost all cell molecules [26–28].

The intensification of peroxidation in healthy residents of industrial area causes the reaction from AOD system. The increasing total antioxidant activity (AOA on 82%) makes it possible to some extent restrain the oxidative modification of macromolecules and maintain the redox potential on a level corresponding to the intense organism response to industrial environment exposure. CuZn-SOD synthesis is increasing (15%) to maintain antioxidant defense, inactivating free radicals and reducing hydroperoxides formation, which leads to decreased GPx (20%). The reduction potential of the thiol-disulfide unit is due to the increasing Trx (33%) and GSH (30%) together with stable reductases levels (GR and TrxR 1).

The results of the correlation analysis between the analyzed processes in healthy residents of the industrial area are shown in Table 6.

The highest level of correlation (−0.84, *p* = 0.0001; Table 6) was found for AOA and MDA. Negative direction of link reflects the restraining effect of AOD systems on the accumulation of lipid peroxidation products at this stage of interaction. Both MDA (−0.54, *p* = 0.022; 0.71, *p* = 0.001) and MDA/AOA (−0.54, *p* = 0.023; 0.71, *p* = 0.0001) have shown correlation with glutathione system parameters. GSH provides the reduction of hydroperoxides in membrane phospholipids via glutathione-S-transferases and GPx, and thereby the activation of glutathione antioxidant system interrupts lipid peroxidation chain reaction [29, 30].

The intensity of protein oxidative modifications is highly affected by the thioredoxine system (−0.58, *p* = 0.011). The thioredoxin system prevents the formation of lipid peroxides conjugating with amino acids in proteins. In addition, TRX restores the disulfide bonds in oxidized proteins. The major role in maintaining the level of MMPL in exposure to microparticles belong to glutathione unit, glutathione peroxidase and glutathione (−0.57, *p* = 0.002; 0.51, *p* = 0.006).

Oxidative DNA damage in healthy residents of industrial area causes an active response from the thioredoxine system (0.63, *p* = 0.004; 0.53, *p* = 0.022) and SOD (0.72, *p* = 0.001). The thioredoxine unit provides DNA integrity protection by maintaining the cell redox potential, signaling to the other AOD units and participating in DNA damage repair. SOD provides the first line of defense against free radical damage to macromolecules due to the inactivation of superoxide anion radicals.

## 4. Discussion

Currently, air pollution is one of the most dangerous environmental risks to public health. According to World Health Organization, air pollution causes 3.7 million deaths per year in world [31]. The microsized particles are the most dangerous to health in industrial air pollution. It has been shown that the microparticles have proinflammatory, carcinogenic, teratogenic, cytotoxic effects and cause allergy [19, 21, 32].

Atmospheric suspension in the park area consists of natural ingredients with which people and animals have come into contact throughout their evolution, particles of minerals and rocks, and organic components (pollen, detritus, insect fragments, and aeroplankton). Technogenic suspensions in the industrial area contain toxic components. The most dangerous technogenic particles are soot and metal microparticles. Particularly noteworthy are the solid particles from diesel exhaust. Soot from transport exhaust is a complex multicomponent system containing not only soot but also a large number of metals, including toxic metals. The capability of soot particles to increase the risk of cancer and cause premature mortality due to respiratory complications and cardiovascular disease was shown previously [33], especially for nanoscale particles [34, 35]. Recent researches on toxicology show that the importance of indicating metals in atmospheric particles suspensions is due to fact that nano- and microparticles of metals and metal oxides have the most evident toxicity [36] and can cause metal allergies [37, 38].

In our study, we determined that the chrome content was higher in snow samples from the industrial area than in the park area. Chrome is capable of causing significant changes in chromatids due to chronic exposure, which indicates its mutagenic and carcinogenic properties. The mechanism of chrome genotoxicity is linked with the inhibition of DNA synthesis and the activation of DNA oxidative damage and apoptosis [39–41]. Recent data indicate the involvement of mismatch repair in DNA double-strand breaks. Mutagenic adducts of ascorbate-chromium-DNA cause double-strand breaks repair errors through nonhomologous end joining [42].

In addition to the direct effects, microparticles cause the induction of oxidative stress, which activates various signaling pathways. Oxidative stress may trigger inflammatory responses in the lungs, circulatory system, or even remote tissues [43]. ROS, as the inducers of oxidative stress, disrupt the functioning of cells and damage their genome [44]. The genotoxic changes affect the genetic expression and the regulation of the inflammation [45]. The thioredoxin system protects cells from apoptosis and maintains the immune cells homeostasis [46].

The parameters of the antioxidant defense system analyzed in our study indicate the negative impact of microparticles on human health. The observed dynamics of the studied processes specify an early stage of ROS damage response. At this stage, oxidative damage affects the most sensitive parameters, the structural and functional state of proteins and leukocyte predisposition to apoptotic changes. ROS excess and the increasing concentration of lipid peroxidation products in the blood contribute to the induction of protein oxidative modification, including irreversible carbonylation [30, 47]. DNA oxidative damage causes the disruption of transcription and the possible synthesis of proteins with altered physical and chemical properties that can change the metabolism and structural/functional cell status. Disorders in catalytic capabilities and protein repair can lead to the activation of apoptosis. The further degradation of proteins can cause necrotic lysis [30, 48].

The character of the disorders related to exposure to finely dispersed atmospheric particles indicates a shift in prooxidant-antioxidant balance. A healthy organism under tolerable oxidative stress conditions can partially restrain its damage level [49]. Specifically, an organism through the activation of natural adaptive reactions can increase the production of antioxidant molecules, thus blocking peroxidation and repairing oxidative stress-induced damage [49, 50]. For example, in our previous studies, we have also reported that although different kind of xenobiotics (e.g., anabolics, insecticides, and pesticides) induced oxidative stress, they also increased antioxidant molecules (e.g., GSH and catalase) as a compensatory mechanism [51–53]. The thioredoxin and glutathione systems selectively control the activity of certain proteins and signal transduction pathways and the thioredoxin system regulates a wider range of targets [54]. However, some proteins are double-regulated by thioredoxin and glutathione both by reduction of a disulfide bond via thioredoxin/glutaredoxins and by glutathionylation of a cysteine [55]. The high activity of antioxidant systems in healthy residents of the industrial area indicates the presence of adaptive reserves, ensuring to some extent the compensation of oxidative modifications.

The glutathione unit mainly contributes to protection; GSH works as a hydroperoxides primary utilizer and SOD supports the static concentration of superoxide radicals at a certain level, thus protecting the cell structures from damage by superoxide and hydroxyl radicals and creating conditions for the normalization of cell energy function and decrease in protein oxidative damage. The AOD thioredoxine unit is activated by increasing oxidative damage as a main reparative and protective factor for DNA and proteins (thiol-disulfide reducing activity).

The processes of adaptation to oxidative stress are likely to be associated with an increase in Н_2_О_2_-degrading activity and its optimal allocation through antiperoxidant enzymes. This is carried out by activating the transcription factor NF-*κ*B [56], increasing antioxidant enzymes gene expression and limiting protein and lipid peroxidation.

## 5. Conclusions

The environmental situation in the studied areas of Vladivostok is characterized by different levels of coarsely and finely dispersed particles in the air and their varying compositions, thus causing different organism responses.

The exposure to finely dispersed particles in atmospheric air causes the oxidative modification of proteins and DNA that contribute to changes in the energy potential of leukocytes in healthy city/industrial seaport residents. The pronounced response by the AOD thiol-disulfide systems maintains the balance of the peroxidation-antioxidation processes protecting cellular and subcellular structures from significant oxidative damage at this stage on level corresponding to the intense organism response to industrial environment exposure. However, long-term, stress-inducing exposure to microsized particles can lead to chronic changes in signal transduction and gene expression, the depletion of the repair and adaptation capabilities of organisms, and the development of pathologies.

## Figures and Tables

**Table 1:** Characteristics of the population studied subjects.

Subjects	Group 1	Group 2
*N*	125	130
Men	58	63
Women	64	70
Age (years), mean ± SD	35 ± 5.6	38 ± 6.1
Smoking	Never	Never
Professional hazard	No	No
Alcohol consumption frequency	Rarely drinking	Rarely drinking

**Table 2:** Analyzed blood parameters and used reagents.

Parameter	Functions	Reagent
Thioredoxin 1 (TRX 1)	Thioredoxin acts as antioxidant by facilitating the reduction of other proteins by cysteine thiol-disulfide exchange and also serves as electron donor for enzymes such as ribonucleotide reductases, thioredoxin peroxidases (peroxiredoxins), and methionine sulfoxide reductases. It is critical for redox regulation of protein function and signaling via thiol redox control.	Thioredoxin-1 ELISA (Northwest Life Science Specialties, LLC, USA)

Thioredoxin reductase 1 (TrxR 1)	Thioredoxin reductases are the only enzymes known to catalyze the reduction of thioredoxin.	Thioredoxin Reductase 1 ELISA (Northwest Life Science Specialties, LLC, USA)

Malondialdehyde (MDA)	Malondialdehyde is a product of lipid peroxidation and prostaglandin biosynthesis. It reacts with DNA to form adducts to deoxyguanosine and deoxyadenosine.	Malondialdehyde Assay (Northwest Life Science Specialties, LLC, USA)

8-Hydroxy-2′-deoxyguanosine (8-OHdG)	8-OHdG is one of the most sensitive biomarkers for oxidative stress. It is formed when DNA is oxidatively damaged by ROS.	8-OHdG ELISA (Northwest Life Science Specialties, LLC, USA)

Protein carbonyl (PC)	Protein CO groups are biomarkers of oxidative stress. They are forming relatively early and have relative stability in comparison with other oxidation products.	Protein Carbonyl ELISA (BioCell Corporation Ltd., New Zealand)

Antioxidant activity (AOA)	Antioxidant activity, or total antioxidant status, is an assessment of the integrated antioxidant system which encompasses all biological components with antioxidant activity.	Total antioxidant assay, Total Antioxidant Status Control (Randox Laboratories Ltd., UK)

Glutathione (GSH)	Glutathione is the major endogenous antioxidant, participating directly in the neutralization of free radicals and reactive oxygen compounds, as well as maintaining exogenous antioxidants such as vitamins C and E.	Glutathione ELISA Kit (MyBioSource, Inc., USA)

Glutathione reductase (GR)	Glutathione reductase catalyzes the reduction of glutathione disulfide to the sulfhydryl form glutathione. Their ration is a key factor in properly maintaining the oxidative balance of the cell. Reduced glutathione reduces the oxidized form of the enzyme glutathione peroxidase.	Glutathione Reductase ELISA Kit (MyBioSource, Inc., USA)

Glutathione peroxidase (GPx)	Glutathione peroxidase reduces lipid hydroperoxides to their corresponding alcohols and free hydrogen peroxide to water.	Glutathione Peroxidase 1 ELISA Kit (MyBioSource, Inc., USA)

Superoxide dismutase (CuZn-SOD)	Superoxide dismutase converts naturally occurring superoxide radicals to molecular oxygen and hydrogen peroxide.	Human Cu/ZnSOD ELISA (eBioscience, USA)

Mitochondrial membrane potential in leukocytes (MMPL)	The evaluation of MMPL allows us to estimate cellular energy state.	MitoProbe JC-1 Assay Kit (Life Technologies, USA)

**Table 3:** Characteristics of areas by objects presented in their territory and their influence on atmospheric suspension composition.

Typical park area		Typical industrial area	
Objects	Influence on atmospheric suspensions	Objects	Influence on atmospheric suspensions
No large industrial enterprises with large-tonnage waste emissions into atmosphere	—	Large industrial enterprises with large-tonnage waste emissions into atmosphere	Sinters, slags, polymetallic particles, glass, synthetic fibers, soot, and black carbon

No heat-electric generating stations	—	Heat-electric generating stations	Coal, sinters, slags, soot, and black carbon

No large road interchanges and highways	—	Large road interchanges and highways	Finely dispersed black carbon and soot, rubber, and metal-containing particles (Fe, Cr, Pb, Zn, Au, Pt, and Ir)

No metalworking and galvanic companies	—	Metalworking and galvanic companies	Metal-containing particles (Fe, Cr, and Zn)

Large forest park zones	Organic detritus	—	—

Riverside or sea coast	Halite, sylvinite, silicates, aluminosilicates, marine organic detritus	—	—

**Coarsely dispersed particles are mostly presented **		**Finely dispersed particles are mostly presented **	

**Table 4:** Parameters of prooxidant system and energy state of cells in healthy residents of the park and industrial areas in city/industrial seaport.

Parameters/group	MDA, *μ*mol/L	MDA/AOA	PC, nmol/mg	8-OHdG, ng/mL	MMPL, %
Group 1 (park area), *N* = 125	2.2 ± 0.03	0.81 ± 0.04	0.42 ± 0.03	7.12 ± 0.07	0.67 ± 0.03

Group 2 (industrial area), *N* = 130	3.1 ± 0.22^*^	1.05 ± 0.15	0.51 ± 0.02^*^	7.98 ± 0.23^*^	1.03 ± 0.05^*^

Note: statistically significant differences between groups at ^*^ *p* ≤ 0.05. MDA: malondialdehyde; AOA: antioxidant activity; PC: protein carbonyl; 8-OHdG: 8-hydroxy-2′-deoxyguanosine; MMPL: mitochondrial membrane potential in leukocytes.

**Table 5:** Parameters of antioxidant system in healthy residents of the park and industrial areas in city/industrial seaport.

Parameters/group	TRX 1, ng/mL	TrxR 1, ng/mL	GSH, *μ*g/mL	GPx, ng/mL	GR, ng/mL	АОА, mmol/L	CuZn-SOD, ng/mL
Group 1 (park area), *N* = 125	6.87 ± 0.08	2.09 ± 0.01	41.59 ± 1.76	0.82 ± 0.01	1.33 ± 0.13	2.1 ± 0.22	13.27 ± 0.20

Group 2 (industrial area), *N* = 130	9.12 ± 0.28^**^	2.27 ± 0.02	54.06 ± 2.81^**^	0.54 ± 0.05^*^	1.38 ± 0.31	3.82 ± 0.06^**^	15.33 ± 0.18^*^

Note: statistically significant differences between groups at ^*^ *p* ≤ 0.05, ^**^ *p* ≤ 0.01. TRX 1: thioredoxin 1; TrxR 1: thioredoxin reductase 1; GSH: glutathione; GPx: glutathione peroxidase; GR: glutathione reductase; AOA: antioxidant activity; CuZn-SOD: superoxide dismutase (CuZn).

**Table 6:** The interrelations of analyzed blood parameters.

Parameters	8-OHdg	TRX 1	PC	TrxR 1	CuZn-SOD	MDA	AOA	MDA/AOA	MMPL	GPx
TRX 1	0.63, *p* = 0.004									

PC		0.52, *p* = 0.027								

TrxR 1	0.53, *p* = 0.022		−0.58, *p* = 0.011							

CuZn-SOD	0.72, *p* = 0.001	0.66, *p* = 0.002								

AOA						−0.84, *p* = 0.0001				

MMPL							−0.55, *p* = 0.02			

GR						0.71, *p* = 0.001	−0.62, *p* = 0.005	0.71, *p* = 0.0001		

GPx		0.54, *p* = 0.021	0.55, *p* = 0.017		0.51, *p* = 0.035				−0.57, *p* = 0.002	

GSH				0.53, *p* = 0.022		−0.54, *p* = 0.022		−0.54, *p* = 0.023	0.51, *p* = 0.006	−0.55, *p* = 0.002

Note: TRX 1: thioredoxin 1; TrxR 1: thioredoxin reductase 1; MDA: malondialdehyde; 8-OHdG: 8-hydroxy-2′-deoxyguanosine; PC: protein carbonyl; AOA: antioxidant activity; GSH: glutathione; GR: glutathione reductase; GPx: glutathione peroxidase; CuZn-SOD: superoxide dismutase (CuZn); MMPL: mitochondrial membrane potential in leukocytes.
